# cAMP receptor proteins in mycobacteria

**DOI:** 10.71150/jm.2607013

**Published:** 2026-10-31

**Authors:** Jeong-Il Oh, Yuna Oh, Yupin Hwang

**Affiliations:** 1 Department of Integrated Biological Science, Pusan National University, Busan 46241, Republic of Korea; 2 Microbiological Resource Research Institute, Pusan National University, Busan 46241, Republic of Korea

**Keywords:** cAMP receptor protein, *Mycobacterium smegmatis*, *Mycobacterium tuberculosis*, regulation of gene expression, transcription factor

## Abstract

Cyclic adenosine monophosphate (cAMP) signaling is a major regulatory system in mycobacteria, and the cAMP receptor protein (CRP) serves as its principal transcriptional effector. In *Mycobacterium tuberculosis*, CRP controls a broad regulon associated with metabolic adaptation, respiration, cell envelope-associated processes, dormancy-related resuscitation, and virulence. Structural and biophysical studies have demonstrated that mycobacterial CRPs differ from the classical *Escherichia coli* paradigm: apo-CRP adopts restrained or asymmetric conformations, whereas cAMP binding promotes a more symmetric, DNA-binding-competent state and enhances the specificity of CRP-DNA interactions. In *Mycobacterium smegmatis*, the major paralog Crp1 (MSMEG_6189) regulates metabolic and respiratory adaptation, oxidative stress defense, and isoniazid susceptibility, whereas Crp2 (MSMEG_0539) appears to play a more limited or subsidiary role. Recent findings further reveal that CRP activity is modulated by lysine acetylation, cooperative promoter recruitment with the virulence-associated response regulator PhoP, and respiratory inhibition-induced increases in intracellular cAMP, thereby linking metabolic and respiratory status to adaptive and virulence-associated gene regulation. This review summarizes the structure, allosteric regulation, regulon, and physiological functions of mycobacterial CRPs, highlighting their role as central integrators of cAMP signaling, respiratory and metabolic state, persistence-associated physiology, and antibiotic susceptibility.

## Introduction

Tuberculosis remains a major global health challenge because *Mycobacterium tuberculosis* can persist for prolonged periods in the host without causing symptoms while adapting to a changing host environment. Among the signaling systems that support this adaptability, cyclic adenosine monophosphate (cAMP), as a second messenger, is particularly prominent. Unlike many bacteria, mycobacteria encode an expanded repertoire of adenylyl cyclases and maintain unusually high intracellular cAMP levels, indicating that cAMP signaling has been adapted for specialized physiological and pathogenic functions (Bai et al., 2011a; Barba et al., 2010; Green et al., 2014; Shenoy and Visweswariah, 2006a, 2006b; Shenoy et al., 2004).

The major transcriptional effector of this pathway is the cAMP receptor protein (CRP), encoded by *Rv3676* in *M. tuberculosis*. Although CRP belongs to the conserved CRP/FNR family, mycobacterial CRP differs from the classical *Escherichia coli* model in both mechanism and regulatory scope (Bai et al., 2005; Gallagher et al., 2009; Green et al., 2014; Kumar et al., 2010; Reddy et al., 2009; Rickman et al., 2005; Stapleton et al., 2010).

Functional studies have established CRP as a global regulator with direct relevance to pathogenic fitness (Rickman et al., 2005). In *M. tuberculosis*, CRP controls a broad regulon associated with metabolic adaptation, respiration, cell envelope-associated processes, redox responses, dormancy-related resuscitation, and virulence, and *crp* disruption compromises growth, survival in macrophages, and virulence in animal models (Akhter et al., 2008; Bai et al., 2005, 2011b; Gazdik and McDonough, 2005; Kahramanoglou et al., 2014; Krawczyk et al., 2009; Liu et al., 2020; Rickman et al., 2005). In *Mycobacterium smegmatis*, the major paralog Crp1 (MSMEG_6189) has emerged as a key regulator of metabolic and respiratory adaptation, oxidative stress defense, and isoniazid (INH) susceptibility, whereas available evidence suggests that Crp2 (MSMEG_0539) plays a more limited role (Aung et al., 2015; Bong et al., 2019; Kim et al., 2025; Ko and Oh, 2020; Ko et al., 2021, 2022; Lee et al., 2014; Sharma et al., 2014). The designations Crp1 and Crp2 have been used inconsistently in the literature. Aung et al. (2015) designated MSMEG_0539 and MSMEG_6189 as Crp1 and Crp2, respectively, based on the numerical order of their locus tags. In contrast, our studies have designated MSMEG_6189 as Crp1, reflecting its greater functional significance. Together, these findings indicate that a conserved CRP-family scaffold has been adapted to distinct ecological and pathogenic demands across the genus.

Structural and biophysical studies showed that apo-CRP can occupy restrained or asymmetric conformations and that cAMP shifts the dimer toward a more symmetric, DNA-binding-competent state (Gallagher et al., 2009; Kumar et al., 2010; Reddy et al., 2009). Additional layers of control arise from metabolically sensitive acetylation, regulatory convergence with PhoP, and respiratory status-dependent changes in intracellular cAMP levels, placing CRP at the intersection of second-messenger signaling, metabolic state, respiration, and virulence control (Di et al., 2023; Jeon et al., 2014; Khan et al., 2022; Ko and Oh, 2020). These advances reposition mycobacterial CRP not simply as a cAMP-binding homolog of the enteric paradigm, but as a specialized global regulator that links environmental sensing to adaptive transcriptional outputs.

In this review, we describe the molecular mechanisms, regulatory logic, and physiological consequences of CRP function in mycobacteria, with particular emphasis on *M. tuberculosis* CRP (CRP_Mt) and the two CRP homologs (Crp1 and Crp2) of *M. smegmatis*. We focus on how CRP integrates cAMP signaling with metabolism, respiration, persistence-associated physiology, and antibiotic susceptibility, and we highlight emerging principles that distinguish mycobacterial CRP from the canonical bacterial CRP model.

## cAMP Signaling in Mycobacteria

In the classical paradigm derived from *E. coli*, cAMP is best known for its role in carbon catabolite repression, where glucose limitation elevates cAMP levels and enables CRP to activate genes required for alternative carbon utilization. More broadly, however, cAMP functions as a bacterial second messenger that influences diverse pathways related to carbon, nitrogen, sulfur, and energy metabolism, as well as virulence-associated processes (Bai et al., 2011a; Green et al., 2014; Haverkorn van Rijsewijk et al., 2011; Shimada et al., 2011).

Mycobacteria represent an especially expanded version of cAMP signaling with multiple adenylyl cyclases: the genomes of *M. tuberculosis*, *M. smegmatis*, *M. avium*, and *M. leprae* encode 15, 10, 12, and 4 adenylyl cyclases, respectively (Shenoy and Visweswariah, 2006a). Mycobacterial adenylyl cyclases respond to diverse cues, including pH, fatty acids, bicarbonate, high ionic strength, hypoxia, and host-derived signals (Abdel Motaal et al., 2006; Agarwal et al., 2009; Bai et al., 2009; Cann et al., 2003; Jeon et al., 2014; Schultz et al., 2025; Shenoy et al., 2005; Tews et al., 2005), thereby generating dynamic cAMP signals that reflect intracellular and environmental conditions. Intracellular cAMP concentrations in mycobacteria have been suggested to reach levels orders of magnitude higher than those in many other bacterial species, likely due to the presence of multiple adenylyl cyclases (Bai et al., 2011a; Dass et al., 2008; Green et al., 2014; Padh and Venkitasubramanian, 1976). This has been proposed to explain why mycobacterial CRPs generally display lower apparent affinity for cAMP than the classical *E. coli* CRP. Moreover, cAMP levels increase during macrophage infection (Agarwal et al., 2009; Bai et al., 2009), and *M. tuberculosis* can deliver cAMP into host cells, thereby modulating host signaling and immune responses (Agarwal et al., 2009; Johnson et al., 2017). The control of cytoplasmic cAMP levels occurs through synthesis by adenylyl cyclases, degradation by cAMP phosphodiesterases such as Rv0805 in *M. tuberculosis*, and excretion; these processes have been thoroughly reviewed by Bai et al. (2011a).

## Discovery and Biochemical Properties of Mycobacterial CRP

The identification of CRP in mycobacteria emerged from genome annotation, comparative sequence analysis, and biochemical characterization of CRP-like transcription factors (Bai et al., 2005; Cole et al., 1998; McCue et al., 2000). In *M. tuberculosis*, Rv3676 was annotated as a CRP homolog based on sequence similarity to *E. coli* CRP, and subsequent studies confirmed that the encoded protein binds cAMP, forms homodimers, and recognizes DNA sequences similar to *E. coli* CRP-binding sites (TGTGA-N_6_-TCACA) (Bai et al., 2005; McCue et al., 2000). These findings established Rv3676 as the major cAMP-responsive transcription factor in *M. tuberculosis* (Table 1).

Subsequent functional studies showed that CRP_Mt acts as a global regulator with broad physiological consequences, including effects on metabolism, respiration, dormancy-related resuscitation, intracellular survival, and virulence (Agarwal et al., 2006; Akhter et al., 2008; Bai et al., 2011b; Knapp et al., 2015; Liu et al., 2020; Rickman et al., 2005). Deletion or depletion of *crp* impairs growth and fitness in vitro, as well as in macrophages and mouse infection models (Bai et al., 2011b; Rickman et al., 2005), establishing CRP as an important determinant of in vivo pathogenic adaptation.

Parallel insights came from studies in *M. smegmatis*, a fast-growing, nonpathogenic mycobacterium that has served as an experimentally tractable comparative model (Sparks et al., 2023). In contrast to *M. tuberculosis*, which carries a single *crp* gene, *M. smegmatis* encodes two CRP homologs, Crp1 (MSMEG_6189) and Crp2 (MSMEG_0539), which share 78% amino acid identity to each other (Table 1) (Aung et al., 2015; Sharma et al., 2014). The genetic locus comprising *crp1* and the downstream putative hydrolase gene *MSMEG_6190* is conserved between *M. smegmatis* and *M. tuberculosis*. In contrast, *crp2* is located at a distinct genomic locus flanked by two regulatory genes (Fig. 1). Sequence homology, conservation of the genetic organization, and comparative biochemical analyses indicate that Crp1 is the principal ortholog of CRP_Mt (Aung et al., 2015; Sharma et al., 2014). Although Crp1 and Crp2 recognize the same consensus motif (TGTGA-N_6_-TCACA), they differ in biochemical behavior and physiological significance. Crp2 binds cAMP with substantially higher affinity than Crp1 (*K*_d_ = ~30 μM for Crp1 and *K*_d_ = ~3 μM for Crp2), but displays weaker DNA-binding activity and much lower cellular abundance (Aung et al., 2015; Ko and Oh, 2020; Sharma et al., 2014). In addition, cAMP binding to Crp1 is entropically driven, as in CRP_Mt, whereas binding to Crp2 is enthalpically driven (Sharma et al., 2014; Stapleton et al., 2010). Aung et al. (2015) compared the transcriptomes of wild-type and *crp2* mutant strains of *M. smegmatis* by microarray analysis. Their findings indicated that Crp2 is involved in regulating genes associated with carbohydrate and glycerol transport, central carbon metabolism, amino acid and polyamine transport, and oxidative phosphorylation. They also showed that the *crp2* mutant grew faster than the wild-type strain in medium supplemented with pentoses such as xylose and arabinose. Expression of the two *M. smegmatis* paralogs is itself embedded in a regulatory circuit: both Crp1 and Crp2 negatively regulate *crp2*, while *crp1* is constitutively expressed (Ko and Oh, 2020). The presence of two CRP homologs in *M. smegmatis* may provide greater regulatory flexibility associated with its saprophytic lifestyle, with Crp1 functioning as the major ortholog and Crp2 serving a subsidiary role (Aung et al., 2015; Ko and Oh, 2020; Sharma et al., 2014). Interestingly, some fast-growing mycobacteria, including *Mycobacterium flavescens*, *Mycobacterium fortuitum*, and *Mycobacterium phlei*, as well as the slow-growing pathogen *Mycobacterium avium*, also encode a Crp2-like homolog in addition to Crp1 (Aung et al., 2015).

## Structural Basis of cAMP-Mediated Allosteric Regulation in CRP_Mt

Earlier studies showed that CRP_Mt differs from *E. coli* CRP in cAMP binding, cAMP-induced conformational changes, and DNA-binding behavior (Bai et al., 2005; Stapleton et al., 2010). CRP_Mt homodimers bind cAMP at their cAMP-binding sites more weakly than *E. coli* CRP (*K*_d_ = ~60 μM for CRP_Mt, *K*_d_ = ~13–16 μM for *E. coli* CRP) (Green et al., 2014; Stapleton et al., 2010). As described above, cAMP binding to CRP_Mt is entropy-driven and noncooperative, in contrast to the cooperative cAMP binding observed in *E. coli* CRP (Stapleton et al., 2010). Moreover, cAMP association with CRP_Mt induces substantially smaller structural rearrangements than those seen in *E. coli* CRP (Gallagher et al., 2009; Kumar et al., 2010; Reddy et al., 2009). Formation of the CRP_Mt-cAMP complex increases DNA binding to a consensus CRP-binding sequence by only approximately twofold, whereas cAMP significantly enhances DNA binding by *E. coli* CRP (Green et al., 2014; Rickman et al., 2005). Thus, apo-CRP_Mt is not completely inactive for DNA binding; rather, cAMP appears to enhance the selectivity and productive geometry of CRP_Mt-DNA interactions.

CRP_Mt preserves the overall architecture of the CRP/FNR family but diverges mechanistically from the classical *E. coli* model (Akif et al., 2006; Gallagher et al., 2009; Kumar et al., 2010; Reddy et al., 2009). Each protomer of CRP_Mt consists of an N-terminal cAMP-binding domain (residues 1–114 of CRP_Mt) and a C-terminal DNA-binding domain (residues 146–223) containing a helix-turn-helix (HTH) motif, connected by an interdomain hinge defined by a central C-helix (residues 117–144) that mediates dimerization and long-range allosteric communication (Gallagher et al., 2009; Kumar et al., 2010; Reddy et al., 2009). Although the global fold is conserved, sequence variation in residues involved in ligand coordination and interdomain coupling between CRP_Mt and *E. coli* CRP has altered the structural and energetic basis by which cAMP regulates DNA binding (Gallagher et al., 2009; Kumar et al., 2010; Stapleton et al., 2010).

Structural studies indicate that apo-CRP_Mt occupies restrained or asymmetric conformations (Gallagher et al., 2009; Kumar et al., 2010). One apo structure revealed a markedly asymmetric homodimer in which the two DNA-binding domains differ in hinge geometry, local packing, and HTH orientation, suggesting that simultaneous productive interaction with DNA is unlikely (Fig. 2A) (Gallagher et al., 2009). Another study emphasized altered interdomain orientation and extensive contacts between the cAMP-binding and DNA-binding domains that appear to stabilize the HTH region in a DNA-incompetent configuration (Fig. 2B) (Kumar et al., 2010). Despite differences in the degree of asymmetry observed, both studies support a common conclusion: in the absence of cAMP, improper interdomain coupling prevents the DNA-binding domains from adopting a productive DNA-binding geometry.

The cAMP-bound structure provides insight into how this restrained state is converted into an active, DNA-binding-competent form (Fig. 2). In the holo state, CRP_Mt adopts an essentially symmetric dimer in which both subunits assume a common conformation compatible with DNA binding (Reddy et al., 2009). Each protomer binds one cAMP molecule in an *anti*-conformation in the N-terminal pocket, and ligand binding remodels the binding cavity while repacking the N-terminal region toward the central C-helix. These changes reposition the C-terminal DNA-binding domains so that the two HTH motifs are arranged symmetrically for productive DNA binding (Reddy et al., 2009). A key mediator of this allosteric transition is the N-domain hairpin (residues 54–73) including the β4 and β5 strands (Reddy et al., 2009). In the apo state, the N-terminal region and hairpin are displaced away from helix C and the DNA-binding module (Gallagher et al., 2009; Reddy et al., 2009). Upon cAMP binding, the N-terminal region repacks toward helix C, and the hairpin establishes a stabilizing network of interdomain interactions with the DNA-binding domain, thereby promoting the active orientation of the HTH motif (Reddy et al., 2009). Normal-mode analyses further suggest that the dominant conformational changes occur mainly across the interdomain interface rather than within individual domains, with hinge-like and spring-like motions enabling domain reorientation (Kumar et al., 2010; Reddy et al., 2009). These findings indicate that allostery in CRP_Mt is distributed across an integrated structural network involving the ligand-binding pocket, the N-domain hairpin, the central C-helix, and the DNA-binding domains. Taken together, current structural evidence supports a model in which mycobacterial CRP samples a conformational landscape of restrained or asymmetric states, and cAMP shifts this equilibrium toward a more symmetric and DNA-binding-competent architecture.

More recent studies indicate that cAMP does not necessarily cause a large increase in affinity for specific promoter DNA. Instead, its principal effect is to suppress nonspecific and higher-order CRP–DNA oligomerization while stabilizing discrete CRP-target DNA complexes (Fig. 2C) (Garate et al., 2021). In the absence of cAMP, CRP_Mt can form higher-order assemblies through interactions with nonspecific DNA or preformed CRP-DNA complexes. cAMP inhibits and reversibly dissociates these oligomers, favoring defined 1:1 CRP-DNA complexes, and a single cAMP molecule per homodimer may be sufficient to induce this shift (Garate et al., 2021). Thus, cAMP primarily enhances the specificity and productive architecture of CRP_Mt-DNA complexes rather than simply increasing overall DNA-binding affinity.

In the case of *E. coli* apo-CRP, the two DNA-binding domains interact to form a rigid body in which the DNA-recognition helices are buried, preventing them from binding to DNA. Binding of cAMP to CRP induces large structural rearrangements that reposition the DNA-binding domains to allow DNA binding (Fig. 2D) (Green et al., 2014). The apo-CRP structure of *E. coli*, in which DNA binding is completely precluded, explains the significant enhancement of DNA binding by cAMP in contrast to CRP_Mt.

## Characterized CRP-Regulated Genes and Their Physiological Relevance in Mycobacteria

Genome-wide mapping of CRP-binding sites has predicted a broad regulatory network in *M. tuberculosis* spanning metabolic, respiratory, stress-adaptive, cell envelope-associated, virulence-related, and dormancy-related functions (Akhter et al., 2008; Bai et al., 2005; Kahramanoglou et al., 2014; Rickman et al., 2005). The preferred CRP-binding sequence is generally represented as TGTGA-N_6_-TCACA, although many validated binding sites deviate from this ideal consensus (Akhter et al., 2008; Bai et al., 2005; Kahramanoglou et al., 2014; Krawczyk et al., 2009; Rickman et al., 2005). Below, we highlight selected well-characterized CRP-regulated genes in *M. tuberculosis* and *M. smegmatis*, focusing on regulatory mechanisms and experimentally supported physiological relevance; these examples are summarized in Table 2.

### Regulation of *rpfA* in resuscitation from dormancy

One of the earliest and most functionally important CRP targets identified in *M. tuberculosis* is *rpfA*, which encodes Resuscitation-Promoting Factor A. CRP directly activates *rpfA* transcription through an upstream CRP-binding site, thereby linking cAMP signaling to resuscitation from dormancy (Rickman et al., 2005). Because Rpf proteins promote the transition from nonreplicative persistence to renewed growth, this CRP–RpfA axis places CRP at the center of latency–reactivation control (Kana et al., 2008; Rickman et al., 2005; Tufariello et al., 2006).

### Regulation of *whiB1*

In *M. tuberculosis*, CRP directly regulates *whiB1*, an essential gene encoding a redox-sensitive transcription factor that contains a nitric oxide-sensitive [4Fe–4S] cluster (Agarwal et al., 2006; Rickman et al., 2005; Smith et al., 2010; Stapleton et al., 2010). CRP binds two CRP-binding sites upstream of the *whiB1* promoter, centered at −58.5 and −37.5, and disruption of *crp* alters *whiB1* transcript levels (Agarwal et al., 2006; Stapleton et al., 2010). Notably, CRP exerts concentration-dependent effects at this promoter, activating transcription when only the upstream site is occupied but repressing expression when both sites are occupied, a condition favored by high cAMP concentrations (Stapleton et al., 2010). Functionally, this regulatory arrangement may allow *M. tuberculosis* to integrate cAMP signaling with nitric oxide- and redox-responsive transcriptional control mediated by WhiB1. Because WhiB1 is essential and can switch between DNA-binding and non-DNA-binding forms depending on the state of its [4Fe-4S] cluster, CRP-dependent modulation of *whiB1* expression is likely to influence bacterial adaptation to host-associated stresses, particularly nitric oxide exposure within macrophages, and may contribute to transcriptional reprogramming during persistence or infection-associated stress responses (Smith et al., 2010; Stapleton et al., 2010).

### Regulation of *serC* in serine biosynthesis

The *serC* gene encodes a phosphoserine aminotransferase involved in serine biosynthesis. In *M. tuberculosis*, CRP binds the intergenic region between *serC* and the divergently transcribed *Rv0885*, and this binding site is required for full expression of both genes in *M. tuberculosis* and *M. bovis* BCG (Bai et al., 2011b). Consistent with these findings, reduced *serC* expression in *crp* mutants contributes to their slow-growth phenotype in vitro, because supplementation with serine or phosphoserine, as well as constitutive *serC* expression, partially restores growth (Bai et al., 2011b). However, *serC* overexpression does not rescue the intramacrophage growth defect of the *M. tuberculosis* *crp* mutant, indicating that CRP controls additional genes required for host adaptation and virulence (Bai et al., 2011b). Thus, regulation of *serC* illustrates how CRP links cAMP-responsive transcription to amino acid biosynthesis and bacterial growth fitness.

### Regulation of succinate dehydrogenase operons in the TCA cycle

CRP directly regulates the *Rv0249c*–*Rv0247c* operon in members of the *M. tuberculosis* complex, including *M. bovis* BCG, which encodes a succinate dehydrogenase (SDH) complex required for optimal growth and virulence (Knapp et al., 2015). This locus is regulated through an unusually complex mechanism involving four CRP-binding sites distributed across the upstream region and within the adjacent *Rv0250c* coding sequence. Functional analyses showed that CRP controls expression from two promoters, one upstream of *Rv0250c* and an internal promoter upstream of *Rv0249c*, and that an intragenic CRP-binding site within *Rv0250c* is required for maximal *Rv0249c* expression (Knapp et al., 2015). Thus, CRP can act both positively and negatively at this locus depending on binding-site and promoter context, highlighting a noncanonical regulatory architecture in which intragenic CRP binding makes a direct and biologically meaningful contribution to operon expression. *M. tuberculosis* and *M. bovis* also contain another operon encoding SDH, *sdhCDAB* (*Rv3316*–*Rv3319*), but CRP_Mt did not bind the upstream region of this locus in vitro (Knapp et al., 2015).

*M. smegmatis* likewise contains two SDH operons, *sdh1CAB* (*MSMEG_0419–MSMEG_0417*) and *sdh2CDAB* (*MSMEG_1672–MSMEG_1669*) (Aung et al., 2015; Kim et al., 2025; Pecsi et al., 2014). Crp1 exerts opposite effects on these systems: it represses *sdh1* while activating *sdh2* (Kim et al., 2025). Accordingly, these isoenzymes have been demonstrated to respond differentially to nutrient limitation, hypoxia, and respiratory inhibition (Aung et al., 2015; Berney and Cook, 2010; Pecsi et al., 2014).

### Negative regulation of acetate metabolism

*M. smegmatis* possesses four acetyl-CoA synthetase genes (*MSMEG_0718, MSMEG_3986*, *MSMEG_5650*, and *MSMEG_6179*) whose products catalyze the formation of acetyl-CoA from acetate, CoA-SH, and ATP via an acetyl-AMP intermediate (Ko et al., 2022). AcsA1 (MSMEG_6179) is the dominant acetyl-CoA synthetase isoform and is required for efficient growth on acetate, establishing it as the major entry point for acetate into central metabolism (Ko et al., 2022). *acsA1* is directly repressed by Crp1 through a defined upstream CRP-binding site (Ko et al., 2022). Loss of *crp1* causes substantial derepression of *acsA1*, and mutation of the CRP-binding site similarly relieves repression, confirming a direct negative regulatory mechanism. Importantly, respiratory inhibition decreases *acsA1* expression, consistent with prior evidence that respiratory stress elevates intracellular cAMP and enhances Crp1 activity (Jeon et al., 2014; Ko and Oh, 2020). Under these conditions, repression of *acsA1* would be expected to limit conversion of acetate to acetyl-CoA, thereby linking respiratory state to carbon flux control.

### Regulation of the glyoxylate shunt

*M. smegmatis* has two isocitrate lyase isozymes (MSMEG_0911 and MSMEG_3706) that catalyze cleavage of isocitrate to succinate and glyoxylate. Isocitrate lyase is a key component of the glyoxylate shunt, which supports growth on fatty acids or acetate as sole carbon sources by enabling anaplerotic assimilation of acetyl-CoA (Ko et al., 2021). Metabolic flux through the glyoxylate shunt is controlled transcriptionally through opposing effects from RamB and Crp1 converging on the major isocitrate lyase gene, *icl1* (*MSMEG_0911*) (Ko et al., 2021). RamB directly activates *icl1* through multiple upstream binding sites, and succinyl-CoA serves as its coactivator. In contrast, Crp1 represses *icl1* during growth on glucose, consistent with the higher intracellular cAMP concentrations measured under glucose relative to acetate conditions. Accordingly, strong induction of *icl1* expression during growth on acetate reflects the combined effects of RamB-mediated activation and relief of CRP-mediated repression, ensuring that the glyoxylate shunt is engaged only under metabolically appropriate conditions. In *M. tuberculosis*, inactivation of *icl* attenuates persistence and virulence in mice and reduces survival in activated macrophages (McKinney et al., 2000).

### Regulation of glycerol catabolism

Glycerol utilization in *M. smegmatis* is governed by a multilayered regulatory system centered on the *glpFKD* operon (*MSMEG_6758-MSMEG_6759-MSMEG_6761*) (Bong et al., 2019). This operon encodes the glycerol facilitator GlpF, glycerol kinase GlpK, and the major glycerol-3-phosphate dehydrogenase GlpD, and thus forms the core transcriptional unit for glycerol catabolism. The *glpFKD* operon is under tripartite control by GylR, Crp1, and SigF (Bong et al., 2019). GylR activates operon expression in response to glycerol metabolism, with glycerol-3-phosphate acting as the effector that weakens GylR binding and remodels its oligomeric state from homotetramer to homodimer, thereby enabling induction. By contrast, Crp1 directly represses the operon, as shown by identification of a CRP-binding site in the promoter-proximal region, specific Crp1 binding to the upstream DNA, and marked derepression of *glpF* in the Δ*crp1* mutant. Because Crp1 is a cAMP-responsive regulator, this repression functionally links glycerol catabolism to intracellular cAMP signaling. At the same time, the alternative sigma factor SigF positively regulates *glpFKD* under respiration-inhibitory and energy-limited conditions: inactivation of *sigF* reduces *glpFKD* expression, whereas respiratory impairment in the Δ*aa*_3_ background (an *aa*_3_ cytochrome *c* oxidase mutant) markedly induces *glpFKD* expression. Thus, Crp1 does not act in isolation but rather as part of an integrated regulatory network in which glycerol availability, cAMP status, respiratory state, and cellular energy limitation are collectively interpreted to determine the extent of glycerol catabolic flux.

### Regulation of oxidative phosphorylation

Crp1 is also involved in regulation of oxidative phosphorylation in *M. smegmatis*. It directly activates the *cydAB* (*MSMEG_3233–MSMEG_3232*) operon, which encodes cytochrome *bd* quinol oxidase, under respiration-inhibitory conditions, particularly when the *bcc*_1_-*aa*_3_ branch of the respiratory electron transport chain (ETC) is impaired (Aung et al., 2014; Ko and Oh, 2020). Two CRP-binding sites centered at −65.5 and −93.5 relative to the transcription start site were identified upstream of the *cydAB* promoter, and these sites are required for induction under respiration-inhibitory conditions in which cellular cAMP levels increase (Aung et al., 2014; Jeon et al., 2014; Ko and Oh, 2020). This response helps maintain electron transport and limits excessive reactive oxygen species (ROS) accumulation when the respiratory chain is compromised. Consistent with this role, loss of Crp1 alters the broader composition of the respiratory ETC, including reduced cytochrome *bcc*_1_ levels, upregulation of the *nuo* and *sdh1* operons (encoding type I NADH dehydrogenase and succinate dehydrogenase, respectively), and downregulation of *sdh2*, without affecting expression of *ndh*, which encodes the major NADH dehydrogenase (Kim et al., 2025). CRP therefore functions as a higher-order regulator of respiratory remodeling in *M. smegmatis*.

### Oxidative stress defense

Crp1 also contributes to oxidative stress defense by regulating *ahpC* (*MSMEG_4891*), which encodes alkyl hydroperoxide reductase (Lee et al., 2014). This enzyme reduces organic peroxides to their corresponding alcohols and hydrogen peroxide to water. In *M. smegmatis*, Crp1 activates *ahpC* expression, and *ahpC* transcript levels are increased in response to elevated cAMP under oxidative stress conditions (Lee et al., 2014). A CRP-binding site is centered at −81.5 relative to the transcription start point of *ahpC*. Because oxidative stress also relieves FurA-mediated repression of *ahpC*, dual control by FurA and Crp1 drives induction of *ahpC* expression under oxidative stress conditions (Lee et al., 2014, 2018).

Collectively, these examples show that mycobacterial CRP regulates diverse promoter architectures and physiological outputs rather than functioning as a narrowly dedicated metabolic regulator.

## Post-translational Regulation: Acetylation Links Metabolism to Transcription

An additional layer of CRP regulation in *M. tuberculosis* is provided by reversible lysine acetylation. CRP itself is acetylated at a conserved lysine residue, K193, located within the C-terminal DNA-binding domain, and this modification has clear functional consequences for transcriptional regulation (Di et al., 2023). Functional analyses showed that acetylation of K193 reduces the DNA-binding ability of CRP and inhibits transcriptional activation of CRP-dependent genes. Consistent with these molecular effects, the acetylation state of K193 influences mycobacterial growth phenotypes, stress adaptation, intracellular survival, and virulence in infection models (Di et al., 2023). These findings indicate that acetylation does not merely fine-tune CRP transcriptional activity, but substantially alters the physiological output of the CRP regulon.

Importantly, CRP acetylation is mechanistically connected to central metabolism through acetyl phosphate (AcP), a high-energy intermediate of the phosphotransacetylase–acetate kinase pathway (Di et al., 2023; Ko et al., 2022). In vitro, AcP acetylates CRP in a dose- and time-dependent manner, and in vivo the level of K193 acetylation correlates with intracellular AcP abundance under conditions that alter carbon flux (Di et al., 2023). Thus, changes in metabolic state can be transmitted directly to the transcriptional machinery through nonenzymatic modification of CRP. This provides a concrete mechanism by which carbon metabolism influences gene regulation in mycobacteria.

The modification is reversible. Acetylated CRP can be deacetylated by the NAD^+^-dependent deacetylase Rv1151c, linking CRP activity to cellular redox and energy status in addition to AcP availability (Di et al., 2023). Notably, K193 acetylation was reported to decrease under several stress or signaling conditions associated with enhanced CRP-dependent responses, including elevated cAMP, acidic pH, oxidative stress, and heat stress, whereas expression of the deacetylase increases under these conditions (Di et al., 2023). Together, these observations suggest that CRP activity is tuned by the combined action of ligand binding, metabolic acetyl donors, and enzymatic deacetylation.

Viewed in this context, acetylation adds a metabolically sensitive post-translational layer to CRP regulation in *M. tuberculosis*. CRP therefore sits at the intersection of second-messenger signaling, metabolic state, and transcriptional control, with K193 acetylation providing a mechanism by which fluctuating intracellular carbon and energy conditions can be translated into altered promoter occupancy and virulence-associated gene expression.

## Regulatory Convergence with PhoP

CRP function in *M. tuberculosis* is further shaped by its integration into other regulatory networks, most notably through convergence with the virulence regulator PhoP of the PhoPR two-component system (Walters et al., 2006). A subset of CRP-controlled genes, including *whiB1*, *icl1*, and *umaA*, also requires PhoP for full expression in *M. tuberculosis*, particularly under host-relevant stress conditions such as acidic pH and nitric oxide exposure (Khan et al., 2022). This effect does not appear to arise from PhoP-dependent control of *crp* transcription. Rather, PhoP promotes CRP occupancy through protein-protein interaction (Khan et al., 2022). Mechanistically, phosphorylated PhoP facilitates CRP recruitment at selected promoters, and both regulators can form a ternary complex on promoter DNA, indicating that transcriptional activation at these loci depends on coordinated promoter occupancy rather than independent parallel regulation. This mode of regulation places CRP within a larger pathogenic network in which cAMP-responsive transcription is integrated with PhoP-dependent virulence signaling.

## Respiratory Inhibition-Associated Activation of CRP-Dependent Transcription

Inactivation of the *aa*_3_-type cytochrome *c* oxidase, which is the major terminal oxidase of the respiratory ETC, was shown to increase intracellular cAMP levels in *M. smegmatis* by 3.2-fold, with an even greater increase observed when aerobically grown wild-type *M. smegmatis* was treated with KCN, an inhibitor of the *aa*_3_ cytochrome *c* oxidase (Ko and Oh, 2020). Consistent with this observation, intracellular cAMP levels in *M. smegmatis* have also been reported to rise significantly under hypoxic conditions (Jeon et al., 2014). Notably, a considerable proportion of genes induced in the Δ*aa*_3_ mutant lacking the *aa*_3_ oxidase relative to the wild-type strain were expressed at lower levels in the Δ*crp1* mutant than in the wild-type strain (Ko and Oh, 2020). Moreover, a substantial subset of genes differentially expressed in *M. tuberculosis* grown under hypoxic conditions, compared with aerobic growth, has been assigned to the CRP regulon (Bai et al., 2005; Sherman et al., 2001). Together, these findings support a model in which respiratory ETC inhibition elevates intracellular cAMP levels and thereby enhances CRP-dependent transcriptional activity, a mechanism best established in *M. smegmatis* and consistent with hypoxic CRP-regulon responses in *M. tuberculosis*. Crp1-mediated induction of the *cydAB* operon under respiration-inhibitory conditions provides a representative example of this regulatory logic (Ko and Oh, 2020).

## CRP and Antibiotic Susceptibility: The Isoniazid Connection

Recent work in *M. smegmatis* has revealed a direct link between CRP function and antibiotic susceptibility. Inactivation of *crp1* markedly increases the sensitivity of *M. smegmatis* to INH, a prodrug activated by the catalase-peroxidase KatG (Kim et al., 2025). This phenotype appears to result from several interconnected changes in oxidative phosphorylation and ROS generation. Compared with the wild-type strain, the Δ*crp1* mutant shows elevated cellular ROS levels, likely due to a bottleneck in electron flow at the *bcc*_1_ complex of the respiratory ETC (Kim et al., 2025). This bottleneck appears to arise from multiple factors, including reduced abundance of the cytochrome *bcc*_1_ complex, increased expression of the *nuo* and *sdh1* operons, and loss of *bd* quinol oxidase induction under conditions of diminished *bcc*_1_ levels (Kim et al., 2025).

In addition to *katG1* upregulation, increased ROS levels in the mutant may enhance INH activation as KatG-mediated INH activation involves peroxide-dependent radical chemistry (Ghiladi et al., 2005; Rosner and Storz, 1994; Zhao et al., 2006). This upregulation of *katG1* results from the inactivation of FurA, a peroxide-sensing transcriptional repressor (Lee et al., 2018). Because inactivation of alkyl hydroperoxide reductase (AhpC) is known to increase INH susceptibility, and Crp1 serves as an activator of *ahpC* expression, loss of Crp1 may further contribute to increased INH susceptibility (Kim et al., 2025; Lee et al., 2014). These findings provide a clear example of how a global transcription factor can influence antibiotic susceptibility through simultaneous control of drug activation, respiratory physiology, and ROS defense. They also raise the possibility that CRP-related pathways may represent useful targets for strategies designed to potentiate antitubercular therapy with INH.

## Conclusion and Future Perspectives

Current evidence establishes CRP as a central regulator that links cAMP signaling to adaptive transcriptional responses in mycobacteria. The physiological outputs of this regulation differ by species and experimental context, ranging from virulence- and dormancy-associated functions in *M. tuberculosis* to metabolic, respiratory, oxidative stress, and INH susceptibility phenotypes in *M. smegmatis*.

CRP activity is shaped by multiple regulatory inputs. In *M. tuberculosis*, cooperation with PhoP integrates CRP into virulence-associated regulatory networks, while lysine acetylation links CRP-dependent transcription to metabolic state. The newly recognized activation of CRP under respiration-inhibitory conditions adds another important layer to this regulatory framework. Respiratory ETC inhibition, including impairment of the *aa*_3_-type cytochrome *c* oxidase, elevates intracellular cAMP levels and enhances CRP-dependent transcriptional responses in *M. smegmatis*. Similar increases in cAMP have also been associated with hypoxic growth and acidic pH. Thus, mycobacterial CRP appears to function as an integrator of cAMP signaling, respiratory status, metabolic state, and stress adaptation.

Future studies should define how cAMP dynamics, CRP allostery, promoter architecture, PhoP cooperation, and acetylation are integrated under physiologically relevant conditions such as hypoxia, nutrient limitation, oxidative stress, and intracellular infection. Given its roles in virulence, persistence-associated physiology, respiration, and drug susceptibility, the CRP regulatory network may provide promising opportunities for interventions that weaken mycobacterial survival or potentiate antibiotic activity.

## Figures and Tables

**Fig. 1.**
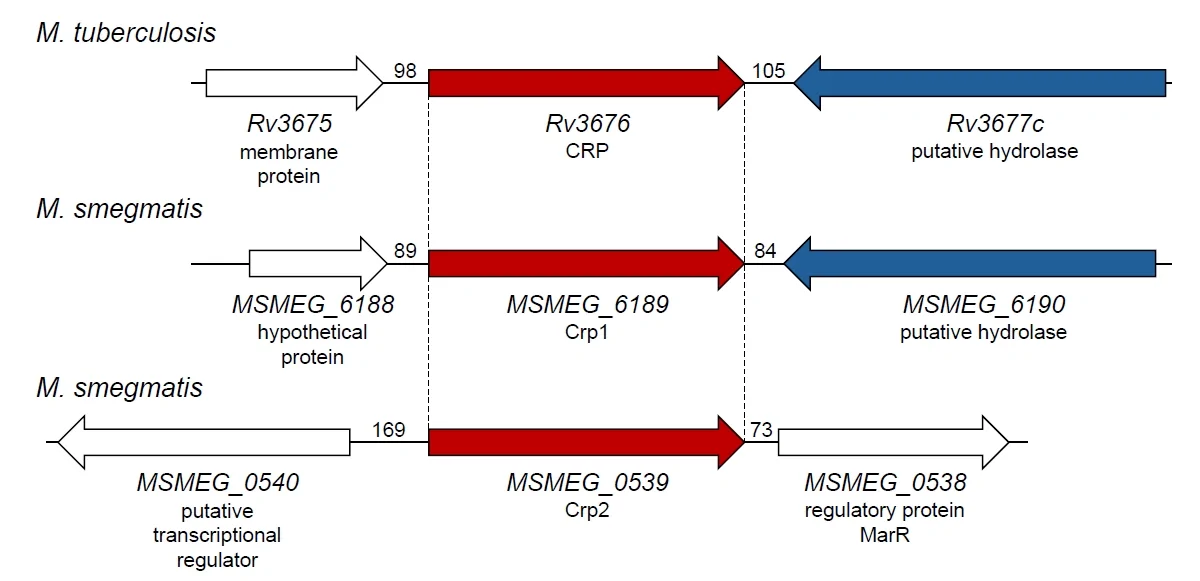
Genetic organization of genes encoding CRP orthologs in *M. tuberculosis* and *M. smegmatis*. The *Rv3676* (*crp*) locus in *M. tuberculosis* H37Rv and the *MSMEG_6189* (*crp1*) and *MSMEG_0539* (*crp2*) loci in *M. smegmatis* mc^2^155 encode CRP orthologs. The downstream genes *Rv3677c* and *MSMEG_6190*, located adjacent to *Rv3676* and *MSMEG_6189*, respectively, are conserved and predicted to encode 264-amino-acid hydrolases. Orthologous genes are indicated using the same color. The numbers between neighboring genes represent intergenic distances in base pairs.

**Fig. 2.**
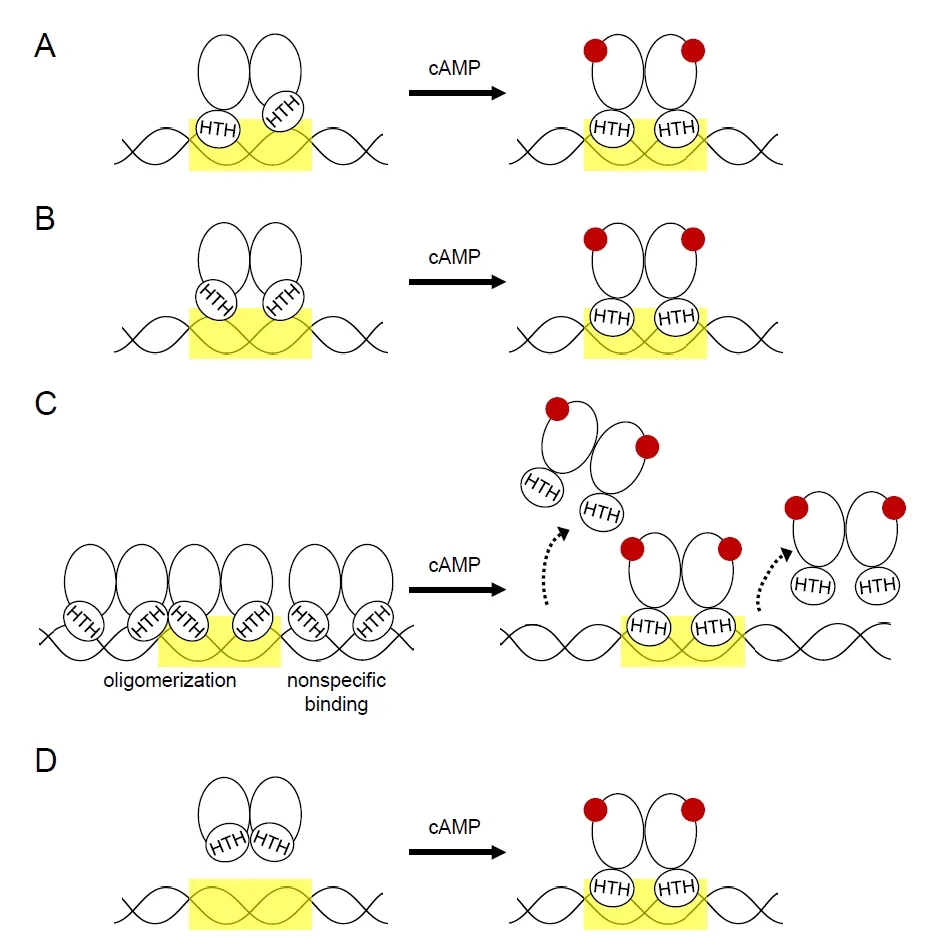
Proposed mechanisms of CRP activation by cAMP binding. (A) In the absence of cAMP, apo-CRP adopts an asymmetric conformation in which the two subunits display distinct orientations of their HTH motifs, thereby disfavoring simultaneous DNA binding by both subunits. cAMP binding stabilizes a symmetric dimeric conformation in which both subunits are properly oriented for DNA recognition. (B) Apo-CRP may retain an overall symmetric dimer architecture; however, altered interdomain arrangements and extensive contacts between the cAMP-binding and DNA-binding domains could restrict the HTH motifs in a DNA-binding-incompetent state. Binding of cAMP relieves these constraints and promotes a conformational state compatible with DNA binding. (C) Apo-CRP may associate with both specific and nonspecific DNA sequences or form higher-order CRP-DNA assemblies. cAMP binding may activate CRP by promoting dissociation from nonspecific DNA or by resolving higher-order CRP-DNA oligomers into discrete 1:1 CRP-DNA complexes. (D) In *E. coli* apo-CRP, the two DNA-binding domains associate with each other to form a rigid structure, causing the DNA-recognition helices to become buried and unable to interact with DNA. Upon cAMP binding to CRP, structural changes occur that reposition the DNA-binding domains into a conformation suitable for DNA binding. Red circles indicate cAMP; yellow boxes indicate CRP-binding sequences.

**Table 1.** Mycobacterial CRP homologs covered in this review article

Species	CRP homolog	Notes
*M. tuberculosis* H37Rv	CRP_Mt/Rv3676	Major cAMP-responsive transcription factor
*M. bovis*	Mb3700	Amino acid sequence identical to CRP_Mt; *crp* genes of *Mycobacterium bovis* BCG strains contain SNPs
*M. smegmatis* mc^2^155	Crp1/MSMEG_6189	Major CRP; more homologous to CRP_Mt than Crp2
*M. smegmatis* mc^2^155	Crp2/MSMEG_0539	Higher cAMP affinity, weaker DNA binding, lower abundance than Crp1; under negative regulation of Crp1 and Crp2

**Table 2.** Representative CRP-regulated genes and associated physiological functions

Gene/locus	Species	Regulation	Function	Reference
*rpfA*	*M. tuberculosis*	Activation	Resuscitation from dormancy	Kana et al. (2008); Rickman et al. (2005)
*whiB1*	*M. tuberculosis*	cAMP concentration- and promoter context-dependent	Redox-sensitive regulator, NO-sensing	Agarwal et al. (2006); Rickman et al. (2005); Smith et al. (2010); Stapleton et al. (2010)
*serC*	*M. tuberculosis*	Activation	Serine biosynthesis / phosphoserine aminotransferase	Bai et al. (2011b)
*Rv0249c–Rv0247c*	*M. tuberculosis*	Activation or repression; promoter context-dependent	TCA cycle / succinate dehydrogenase	Knapp et al. (2015)
*sdh1CAB*	*M. smegmatis*	Repression by Crp1	TCA cycle / succinate dehydrogenase	Aung et al. (2015); Kim et al. (2025)
*sdh2CDAB*	*M. smegmatis*	Activation by Crp1	TCA cycle / succinate dehydrogenase	Aung et al. (2015); Kim et al. (2025)
*acsA1*	*M. smegmatis*	Repression by Crp1	Acetate assimilation / major acetyl-CoA synthetase	Ko et al. (2022)
*icl1*	*M. smegmatis*	Repression by Crp1	Glyoxylate shunt / major isocitrate lyase	Ko et al. (2021)
*glpFKD*	*M. smegmatis*	Repression by Crp1	Glycerol catabolism	Bong et al. (2019)
*cydAB*	*M. smegmatis*	Activation by Crp1	ETC / cytochrome *bd* quinol oxidase	Aung et al. (2014); Ko and Oh (2020)
*ahpC*	*M. smegmatis*	Activation by Crp1	Peroxide defense / alkyl hydroperoxide reductase	Lee et al. (2014)

The table summarizes selected experimentally characterized CRP-regulated genes or loci discussed in the text, indicating the species in which regulation has been examined, the regulatory mode, and the associated biological function. Because many CRP-associated phenotypes have been characterized in species-specific experimental contexts, the table is intended as a functional summary of representative targets rather than a direct one-to-one comparison between *M. tuberculosis* and *M. smegmatis*.
